# Platelet Depletion is Effective in Ameliorating Anxiety-Like Behavior and Reducing the Pro-Inflammatory Environment in the Hippocampus in Murine Experimental Autoimmune Encephalomyelitis

**DOI:** 10.3390/jcm8020162

**Published:** 2019-02-01

**Authors:** Pece Kocovski, Xiangrui Jiang, Claretta S. D’Souza, Zhenjiang Li, Phuc T. Dang, Xiaowei Wang, Weisan Chen, Karlheinz Peter, Matthew W. Hale, Jacqueline M. Orian

**Affiliations:** 1 Department of Psychology and Counselling, School of Psychology and Public Health, La Trobe University, Bundoora, VIC 3086, Australia; 15351260@students.latrobe.edu.au (P.K.); m.hale@latrobe.edu.au (M.W.H.); 2 Department of Biochemistry and Genetics, La Trobe Institute for Molecular Science (LIMS), La Trobe University, Bundoora, VIC 3086, Australia; x14jiang@students.latrobe.edu.au (X.J.); cdsouza@ausbiotech.org (C.S.D.); zhenjiangemail@gmail.com (Z.L.); timothydvl@gmail.com (P.T.D.); weisan.chen@latrobe.edu.au (W.C.); 3 Atherothrombosis and Vascular Biology, Baker Heart and Diabetes Institute, Melbourne, VIC 3004, Australia; xiaowei.wang@baker.edu.au (X.W.); karlheinz.peter@baker.edu.au (K.P.); 4 Department of Medicine, Monash University, Melbourne, VIC 3800, Australia

**Keywords:** platelets, neuroinflammation, EAE, EPM, hippocampus, MS, anxiety-like behavior, C57BL/6, autoimmune disease, MS therapy

## Abstract

The neuropsychiatric symptoms of multiple sclerosis (MS), such as anxiety and depression, can result from disease activity itself as well as psychological reaction to an unfavorable diagnosis. Accordingly, the literature reports evidence of increased anxiety-like behavior in experimental autoimmune encephalomyelitis (EAE), an accepted MS model. Due to the recently described critical role of platelets in inflammation and autoimmune disease, we examined the relationship between platelets, inflammation, and anxiety-like behavior in EAE. In the elevated plus maze, EAE-induced C57BL/6J mice showed decreased time spent in the open arms relative to vehicle-only controls, demonstrating an increase in anxiety-like behavior. This effect occurred in the presence of platelet–neuron association, but absence of lymphocytic infiltration, in the hippocampal parenchyma. Platelet depletion at the pre-clinical disease stage, using antibody-mediated lysis prevented the EAE-induced increase in anxiety-like behavior, while no significant difference in distance moved was recorded. Furthermore, platelet depletion was also associated with reduction of the pro-inflammatory environment to control levels in the hippocampus and prevention of EAE disease symptomology. These studies demonstrate the high efficacy of a platelet-targeting approach in preventing anxiety-like symptoms and clinical manifestations of EAE and have implications for the treatment of neuropsychiatric symptoms in MS.

## 1. Introduction

Multiple sclerosis (MS) is the most common chronic inflammatory central nervous system (CNS) disorder and the leading cause of non-traumatic neurological disability seen in the young adult population [1]. It is manifested by a wide range of symptoms including visual impairment, difficulties with balance, muscle weakness, bladder and bowel dysfunction, and cognitive and emotional problems [2]. Historically, these symptoms have been associated with the presence of focal demyelinating lesions predominantly in white matter [3]. Lesion formation is associated with infiltration of T cells, autoreactive to antigens located in the myelin sheath, with concomitant loss of blood–brain barrier function, leading to the recruitment of other inflammatory effector cells such as monocytes/macrophages and activation of resident microglia [4]. This results in the generation of complex inflammatory cascades which initially induce oxygen and nitrogen reactive species, hypoxia, cytokine/chemokine, and glutamate accumulation; leading to oxidative stress, demyelination, mitochondrial damage, calcium influx, and apoptosis [5].

However, the understanding of MS inflammatory mechanisms is far from complete, with for example the introduction of platelets as new players in the early inflammatory response [6,7]. Platelets are small anuclear blood cells typically associated with vascular homeostasis and blood disease [8]. Recent developments have highlighted a non-homeostatic role for these elements in inflammatory conditions in general [9,10,11,12] and both MS [13] and murine experimental autoimmune encephalomyelitis (EAE) [12,13,14,15], an induced autoimmune-mediated inflammatory CNS disease and an accepted model of MS [16,17]. Thus, considerable evidence for platelet abnormalities and significant differences in levels of platelet products in MS patients has been accumulating [18,19,20,21]. Work by Langer et al. [22] revealed the presence of platelets in MS lesions and (Myelin Oligodendrocyte Glycoprotein) MOG_35–55_-induced-EAE and first established that platelets contribute to the pathogenesis of EAE by promoting CNS inflammation. Subsequently, a link between platelets and lymphocytic activation in early EAE was demonstrated [23] with evidence that platelet degranulation results in the release of soluble factors, including serotonin (5HT) and platelet factor 4 (PF4), which stimulate T cell differentiation towards a pathogenic phenotype. Additionally, a potential platelet-related neurodegenerative mechanism [24] was suggested by the demonstration that platelets form associations with neurons as well as astrocytes, via sialated gangliosides within lipid rafts, resulting in the release of proinflammatory cytokines from platelets, immune cascades, and EAE development. More recent studies by our own group [25] identified platelet accumulation in the circulation in pre-clinical EAE and showed that the timing of this accumulation clearly preceded the earliest identifiable inflammatory cell extravasation. Platelet accumulation was immediately followed by entry into the parenchyma and association with neurons in the spinal cord and retina [25]. Furthermore, a cause and effect relationship between platelet depletion and absence of clinical disease was identified, thereby establishing a driving rather than exacerbating role for platelets in neuroinflammation.

One of the major unanswered questions in MS pathophysiology is the striking difference in lesion development between white matter, characterized by perivascular inflammatory infiltrates and grey matter which is associated with paucity of infiltrating immune cells [26]. This is true of multiple CNS regions, including the cerebral cortex, thalamus, hypothalamus, basal ganglia, hippocampus, cerebellum, and spinal cord [27,28,29,30,31,32,33,34]. The observations of extensive hippocampal demyelination [32] coupled with significant decreases in synaptic density together with reduced expression of proteins specific for neuronal function including memory and learning are of interest, because of the association of this structure with emotion and cognition [35] and the evidence of depression and cognitive defects in MS patients [36,37,38]. Indeed, the significance of these symptoms as primary manifestations of the disease rather than consequences of living with a chronic disease has gained momentum over the last decade [39,40]. Prior investigations of hippocampal pathology in MOG_35–55_-induced EAE in our laboratory [41] revealed a complex pattern of damage, with minor inflammation in the dorsal hippocampus, moderate inflammation in the ventral region of the structure, but severe inflammation in the fimbrium region. Microglia reactivity and CNS stress (identified by αB-crystallin) were prevalent throughout. Taken together, data showed involvement of this structure in EAE as well, with grey matter sub-regions characterized by reduced inflammation relative to white matter sub-regions.

EAE has been suggested as a model for depressive-like behavioral syndrome due to MS [42]. Given the evidence of early symptoms indicative of hippocampal impairment in MS and of pre-clinical entry of platelets in the CNS in EAE, we hypothesized that platelet infiltration into the hippocampus is associated with emotional and cognitive impairment in neuroinflammation and that platelet inhibition will ameliorate these symptoms. Specifically, we performed the elevated plus maze (EPM) test, which is a validated quantifiable test of anxiety-like behavior in mice [43]. These functional investigations were performed in the presence or absence of platelet depletion and further supported by immunochemical and molecular analyses.

## 2. Experimental Section

### 2.1. EAE Induction

C57BL/6J mice were obtained from the Animal Resource Centre (Perth, Australia) and housed under standard conditions at 23 °C and 12:12 light:dark cycle, on standard rodent chow (Barastoc, Rat and Mouse Feed, Ridley AgriProducts, Harristown, QLD, Australia) with food and water ad libitum [44]. All procedures were approved by the institutional Animal Ethics Committee and performed strictly in accordance with regulations set by the National Health and Medical Research Council of Australia. Only female mice aged 12–16 weeks were used in experimentation, due to the well-documented differences in disease incidence (3F:1M) and clinical sub-types (relapsing–remitting clinical course being more common in women) in MS [44]. MOG_33–55_-induced EAE was performed as previously described [45]. Briefly, on day 0 mice received two subcutaneous injections, each containing 100 µg MOG_35–55_ peptide in 100 μL of phosphate buffered saline (PBS; 0.01 M phosphate, 15 mM NaCl, pH 7.4), in an equal volume of complete Freund’s adjuvant (Sigma-Aldrich, St. Louis, MO, USA) supplemented with 4 mg/mL of *Mycobacterium tuberculosis* (Becton Dickinson, Franklin Lakes, NJ, USA). On days 0 and 2, mice received an intraperitoneal injection of 350 ng of pertussis toxin (PTx) (Sigma-Aldrich) in PBS. Clinical scores were given to monitor disease progression, as follows 0 = no symptoms, limp tail = 1, hind limb weakness = 2, hind limb paralysis = 3, ascending paralysis = 4, and moribund = 5 [45]. Control groups included vehicle-only (VO; omission of MOG_33–55_) and normal mice.

### 2.2. Estimation of Platelet Numbers and Platelet Depletion

Platelet counts were obtained from 50 to 100 µL of blood collected from the submandibular vein into K_2_EDTA-coated blood Microtainers (Becton-Dickinson (BD), Franklin Lakes, NJ, USA), using a Sysmex XS-1000i (Sysmex America Inc. Mundelein, IL, USA) automated hematology analyzer. Platelet depletion (PD) with a polyclonal anti-GPIb alpha (CD42b) preparation (R300, Emfret Analytics, Eibelstadt, Germany) was achieved by IV administration, at seven days post induction (dpi) of EAE and at 0.5 µg/g body weight in 100 μL of phosphate buffered saline (PBS, containing 10 mM phosphate and 150 mM NaCl, Ph 7.4). Alternatively, as control, platelet depletion antibody was administered to vehicle-only mice. Platelet depletion was maintained by repeating the treatment every 48 h. An isotype antibody preparation (C301, Emfret Analytics) was administered to EAE-induced or vehicle-only groups as control, at the same times and dose. In all experiments, *n* = 6 mice/group/time point.

### 2.3. EPM Test

Behavioral testing was performed during daytime, with *n* = 8 mice/group. The EPM consists of a central platform (5 × 5 cm) with four branching arms (30 × 5 cm each) at right angles to each other, where one pair of opposite arms is walled and the other open [46]. Following a single administration of platelet depleting antibody at 7 dpi, the test was conducted at 9 dpi in a soundproof room under dim red lighting (40–41 lux) as previously described [44]. Behavior was recorded using a high definition (HD) webcam connected by a personal computer (PC), by an investigator blinded as to mouse identity and treatment conditions.

### 2.4. Intracellular Cytokine Staining (ICS)

Following humane killing, mice taken from 9 to 16 dpi were exsanguinated by transcardiac perfusion with PBS and lymph nodes, spleen, blood, brain, and spinal cord immediately collected and homogenized for the preparation of singe cell suspensions as described [47]. Briefly, following isolation by Percoll gradient centrifugation, lymphocytes were stimulated by incubation with MOG_35–55_, or proteolipid protein (PLP) 139–151 as control peptide, in the presence of the Golgi inhibitor Befreldin A for 3 to 4 h and subsequent immunostaining with anti-CD4, anti-CD8 and anti-IFN-γ. Sample cells were then counted by flow cytometer (FACSCanto II, BD Biosciences, Franklin Lakes, NJ, USA). Parameters were adjusted by running single marker labeled and negative controls. Events data were exported to .fcs file and analyzed with FlowJo (7.6.2, FlowJo LLC, Ashland, OR, USA). Total population and percentage of cells of interest were processed using Microsoft Excel 2011 and Prism (5.0b, GraphPad Software, Inc, La Jolla, CA, USA). In all experiments, *n* = 6 mice/group/time point.

### 2.5. RNA Isolation, cDNA Synthesis, and qPCR Analysis

Following transcardiac perfusion with PBS, the whole brain was removed and the region containing the dorsal hippocampus (approximately −0.94 to −3.88 mm bregma) was sectioned using a brain matrix (Ted Pella Inc., Redding, CA, USA), with *n* = 4 mice/group. The dorsal hippocampus was collected from both hemispheres using a biopsy punch, 1.5 mm in diameter. RNA was extracted from hippocampal tissue via the Isolate II RNA Mini Kit RNA (BIO-52072, Bioline, Boston, MA, USA) as recommended by the manufacturer and the quality of RNA preparations verified on a microchip electrophoresis system for DNA/RNA analysis, MCE-202 (MultiNa, Shimadzu Corporation, Kyoto, Japan). This was followed by cDNA synthesis using the Tetro cDNA Synthesis Kit (BIO-65043, Bioline), as recommended by the manufacturer. Primer pairs for target genes and reference genes (HSP90, GAPDH and β-actin) and are shown in Table 1.

Quantitative real-time PCR reactions were run on a CFX96 real-time system (Bio-Rad, Hercules, CA, USA). Amplification reactions were performed using RT2 SYBR green qPCR master mix (QIAGEN, Hilden, Germany) and 1 µM primers in a 10 µL volume. All reactions began with initial activation at 95 °C for 10 min, followed by 45 cycles of 95 °C for 15 s, 59 °C for 20 s, and 72 °C for 15 s and a final extension step at 72 °C for 7 min. The cycle threshold (Ct) (the number of cycles for the fluorescent signal to cross the threshold; i.e., above background) values were generated by the Bio-Rad CFX Manager software (version 3.1) and imported into Microsoft Excel 2016 (Microsoft Corporation, Redmond, WA, USA) for conversion to fold expression relative to unit mass (1µg total RNA).

### 2.6. Histology and Immunochemistry

Transcardiac perfusion was performed first with PBS, then 4% *w*/*v* paraformaldehyde. For histological evaluations (*n* = 4 mice/group), tissues were processed for paraffin embedding and sectioned at 7 µm for hematoxylin and eosin (H & E) staining using a standard protocol. Images were captured with a Nikon Ti Eclipse (Nikon, Japan) and camera lucida images were generated, then analyzed using ImageJ (NIH; Bethesda, MD, USA). Data are expressed as lesion area as a % of total area. Alternatively, for immunofluorescence staining, brains were cut in the coronal plane immediately caudal to the medial mammillary nucleus, into forebrain and hindbrain blocks, using a mouse brain matrix (TED Pella Inc. Redding, CA, USA). Each block was then snap frozen by submerging for 5 s into liquid isopentane (Sigma-Aldrich) cooled to approximately −80 °C with dry ice, and stored at −80 °C prior to sectioning. Brains were sectioned coronally at 30 µm and sections stored in cryoprotectant solution [48]. Immunostaining of hippocampal sections was performed as described using polyclonal antibodies to CD3 (at 1:300, Dako, Glostrup, Denmark), ionized calcium-binding adapter molecule 1 ((Iba1) at 1:200, Wako Chemical Industries, Japan) and CD42b, a platelet-specific subunit of the glycoprotein (GP) 1b-V-IX complex mediating platelet adhesion to injured and inflamed vascular surfaces (at 1:100, Emfret Analytics) and a monoclonal antibody against MAP2 (at 1:500 Novus Biologicals, Centennial, CO, USA). Detection was achieved with Alexa 488 or 594 secondary antibodies (Thermo Fisher Scientific, Waltham, MA, USA) and nuclei were stained with 0.001% 4′,6 diamidino-2-phenylindole (DAPI). Images were detected on a Zeiss LSM 780 confocal microscope and images captured using the Zen 2011 (version 2.0.15, Carl Zeiss AG, Oberkochen, Germany) software. Following acquisition of fluorescence intensity (in arbitrary units), quantification of immunofluorescence was performed using ImageJ with *n* = 4 in all groups, with six sections/mouse as described [45].

### 2.7. Quantification of Plasma Soluble P-Selectin (sP-Selectin) by Enzyme-Linked Immunosorbent Assay (ELISA)

Plasma was prepared from blood collected from vehicle-only, vehicle-only/platelet depleted, EAE-induced/isotype antibody-treated and EAE-induced/platelet depleted groups, with *n* = 4 mice/group. The concentration of sP-selectin was determined using a mouse P-selectin ELISA Kit (Thermo Scientific, Frederick, MD, USA), as recommended by the manufacturer and expressed as ng/mL from the standard curve generated in the assay.

### 2.8. Statistical Analyses

Behavior parameters were analyzed using Ethovision XT (version 13, Novus Information Technology, Wageningen, The Netherlands and data analyzed by 2 (treatment:vehicle-only vs. EAE) × 2 (intervention:none vs. platelet depletion) univariate analyses of variance (ANOVA) using the Statistical Package for the Social Sciences (SPSS, v22, IBM, Armonk, NY, USA) for the assessment of group differences between conditions. That is, an interaction effect was examined between the treatment cohort and the intervention cohort, and main effects were also examined. A normality of distribution and homogeneity of variance was assumed and confirmed by a Shapiro–Wilk test (*p* > 0.05) and Levene’s test (*p* > 0.05), respectively. Normal mice were not included in the behavioral test, as it was previously shown that there was no a significant difference between the normal and VO groups in terms of behavior [44]. Post-hoc tests using Fisher’s protected least significant differences (LSD) were performed for all variables when appropriate. The mean difference is significant at the 0.05 level. The *n* = value was 8 mice/group. All other data were analyzed using a two-tailed Student’s *t*-test and shown as mean ± standard error of the mean (SEM).

## 3. Results

### 3.1. There Is a Direct Relationship between Platelet Accumulation and That of Antigen-Specific T Cells in Neuroinflammation

**Figure 1:**
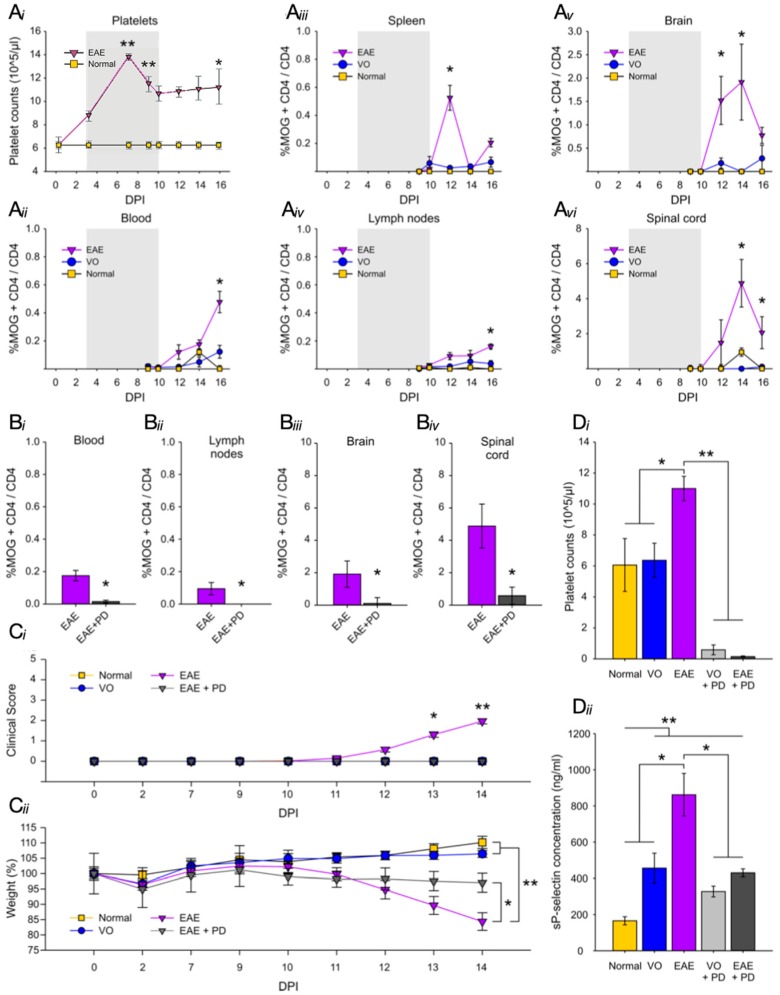
The relationship between platelet accumulation, MOG_35–55_-CD4^+^ cell accumulation and EAE development. (**A**) Platelet counts were performed over the disease course in normal (yellow) and EAE-induced mice (purple) using an automated hematology analyzer and data expressed as platelets numbers/L. (**Ai**) shows significant (** = *p* < 0.01) elevation by 7 dpi, and significantly (* = *p* < 0.05) elevated numbers until 16 dpi. Antigen-specific T cell accumulation was estimated by ICS over the same time course and expressed as % MOG_35–55_-CD4^+^ over total CD4^+^ cells. Comparison between (**Ai**) and (**Aii**–**Avi**) shows a distinct delay between the peak of platelet accumulation in the circulation (shaded area) and that of MOG_35–55_-CD4^+^ cells in blood (**Aii**), secondary lymphoid organs (spleen: **Aiii** and lymph nodes: **Aiv**) and CNS tissues (brain: **Av** and spinal cord: **Avi**). EAE = EAE-induced (purple), VO = vehicle-only (blue) and N = normal (yellow) mice. In both experiments, *n* = 6/group/time point. (**B)** shows inhibition of MOG_35–55_-CD4^+^ cell accumulation by platelet depletion in blood (**Bi**), lymph nodes (**Bii**), brain (**Biii**) and spinal cord (**Biv**). Depletion was initiated at 7 dpi and ICS performed at 14 dpi, with *n* = 6/group. (**Ci)** shows the effect of platelet depletion on disease course, with only EAE-induced/isotype antibody-treated group (purple) exhibiting significantly different clinical scores and weight loss, relative to normal (yellow), VO/PD (blue) and EAE/PD-treated (black) groups. The EAE-induced/isotype antibody-treated group exhibited significant weight loss relative to normal and vehicle only groups and significant weight loss relative to the EAE-induced/PD-treated group (**Cii**). (**Di)** shows confirmation of platelet depletion with anti-CD42b and (**Dii**), estimation of sP-selection by ELISA, showing significantly elevated levels of this component in VO mice, significantly levels in EAE mice and restoration to VO levels by platelet depletion. Experiments **B** to **D** were performed on the same groups of mice. EAE = experimental autoimmune encephalomyelitis, ICS = intracellular cytokine staining, VO = vehicle-only, PD = platelet depletion, CNS = central nervous system, ELISA = enzyme-linked immunosorbent assay.

Platelet numbers were estimated over the disease course in EAE-induced mice (Figure 1Ai) and exhibited an increase from 3 dpi, which reached a peak between 5 and 7 dpi, significantly elevated (*p* < 0.01) above those of normal mice. Subsequently, a partial reduction in platelet numbers was observed, but these remained significantly above control levels (*p* < 0.05) for the remainder of the disease course. Concurrently, accumulation of MOG_35–55_-specific T cells (expressed as the percentage of MOG_35–55_-CD4^+^/total CD4^+^ cells) was estimated by ICS in blood, spleen, lymph nodes, brain, and spinal cord (Figure 1Aii–Avi) in normal, vehicle-only, and EAE-induced mice. In normal and vehicle-only groups, no MOG_35–55_-CD4^+^ cells were ever detected over the time course examined, in any tissue, as expected. In EAE-induced groups, in all of the tissues sampled, the earliest evidence of MOG_35–55_-CD4^+^ cells was between 10 and 12 dpi, namely at least three to six days following the peak of platelet accumulation. Antigen-specific T cell accumulation displayed a monophasic pattern over the disease course in the spleen, brain, and spinal cord, with a peak at 12 dpi (spleen), or 14 dpi (brain and spinal cord), but continued to slowly accumulate in the blood and lymph nodes.

**Figure 2:**
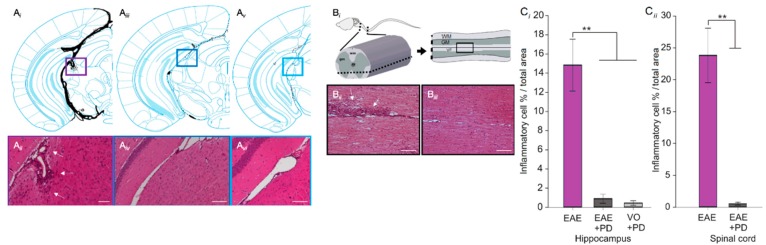
Effect of platelet depletion on inflammatory infiltration into the CNS. (**A**) shows representative H & E stained sections of the effect of platelet depletion in the hippocampus, with camera lucida images to map the lesion locations (**Ai**, **Aiii**, **Av**). **Ai** and **Aii** = EAE-induced/isotype control, **Aiii** and **Aiv** = EAE-induced/PD-treated, **Av** and **Avi** = VO/PD-treated. (**B**) shows representative images of the spinal cord, with **Bi** = region of interest, **Bii** = EAE-induced/isotype control, **Biii** = EAE-induced/PD-treated. Scale bar = 150 µm. (**C**) Quantification of inflammation was performed on *n* = 4 mice/group × 4 sections/mouse, using ImageJ. Data are expressed as lesion area as a % of total area. EAE = experimental autoimmune encephalomyelitis, VO = vehicle only, PD = platelet depletion, WM = white matter, GM = grey matter, VF = ventral funiculus.

To determine whether a direct relationship exists between platelet and MOG_35–55_-CD4^+^ accumulations, ICS was repeated in the presence of platelet depletion, which was induced from 7 dpi using an antibody against CD42b (or GP1bα). In our hands [25], this approach results in reduction in platelet numbers in all control and experimental groups by above 96%, as well as maintenance of low platelet numbers by repeated anti-CD42b administration every 48 h. Evaluation of MOG_35–55_-CD4^+^ accumulation at 14 dpi, showed significant reduction in blood, lymphoid organs and CNS tissues (Figure 1Bi–Biv) in the EAE-induced/platelet depleted group, relative to EAE-induced/isotype antibody-treated group. This group did not develop disease symptoms, as demonstrated by absence of clinical scores by experimental end point, whilst their isotype antibody-treated counterparts reached a mean clinical score of 2.25 ± 1.75 (Figure 1Ci). Some weight loss was observed in EAE-induced/platelet depleted mice, but this was reduced relative to isotype antibody-treated mice (Figure 1Cii). Confirmation of effective platelet depletion in these experiments was provided by the reduced platelet numbers in the EAE-induced/platelet depleted group relative to the isotype antibody-treated controls (Figure 1Di; Supplementary Table S1), together with reduction in levels of sP-selectin, a major marker of platelet activation. Determination of sP-selectin levels by ELISA showed elevation in vehicle-only groups relative to normal mice, presumably from components of adjuvants used in disease generation, further augmented in EAE induced/isotype antibody-treated mice (Figure 1Dii). These levels were restored to those of the vehicle-only group by platelet depletion. Finally, no inflammatory infiltration was detectable in EAE-induced/platelet depleted animals by H&E histological staining of brain (Figure 2A) or spinal cord (Figure 2B) by experimental end point, while severe inflammation was present throughout the whole of the neuraxis in isotype antibody-treated counterparts.

### 3.2. Platelet Depletion Significantly Reduces Anxiety-Like Behavior in EAE-Induced Mice

**Figure 3:**
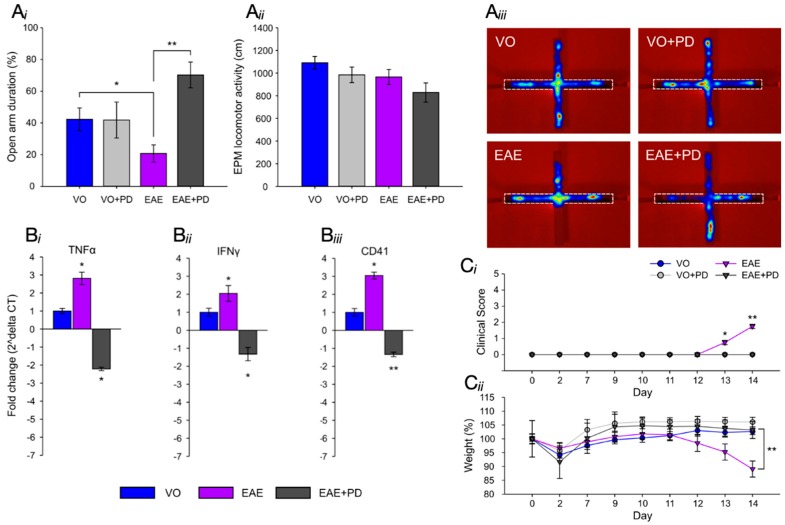
Effect of platelet depletion on anxiety-like behavior and the pro-inflammatory environment in the hippocampus. (**A**) EPM evaluation (*n* = 8/group). (**Ai**) of anxiety-like behavior in control (VO (blue), VO/PD-treated (grey), EAE-induced/isotype antibody-treated (purple) and EAE-induced/PD-treated groups (black), showed no significant difference between VO/isotype antibody-treated versus VO/PD-treated groups, a significant difference (* = *p* < 0.05) between VO and VO/PD-treated versus EAE-induced/isotype antibody-treated groups and a significant difference (** = *p* < 0.01) between EAE-induced/isotype antibody-treated versus EAE-induced/PD-treated groups. The difference between the EAE-induced/PD-treated groups and VO/PD treated groups is not significant. There was no significant difference between any of the groups for total distance moved (**Aii**). (**Aiii**) shows the EPM and representative heat maps from VO, VO/PD-treated, EAE-induced/isotype antibody-treated and EAE-induced/PD-treated groups, with the dotted line showing the closed arm. (**B**) (*n* = 4/group) shows the qPCR analysis of pro-inflammatory cytokines TNF-α (**Bi**) and IFN-γ (**Bii**) and platelet marker CD41 (**Biii**) in VO/PD-treated (blue), EAE-induced/isotype antibody-treated (purple) and EAE-induced/PD-treated (black) groups with significantly elevated TNF-α and IFN-γ and CD41 in the EAE-induced/isotype antibody-treated group relative to the VO/PD-treated group and significant reduction of all markers in the EAE-induced/PD-treated group. (**C**) shows the disease profile in mice used in the EPM test, with only the EAE-induced/isotype control-treated group exhibiting clinical disease (**Ci**) and significant weight loss (**Cii**) by 14 dpi. EPM = elevated plus maze, EAE = experimental autoimmune encephalomyelitis, VO = vehicle only, PD = platelet depletion.

Anxiety-like behavior was evaluated in the EPM as previously described [44], using vehicle-only/isotype antibody-treated, vehicle-only/platelet depleted, EAE-induced/isotype antibody-treated and EAE-induced/platelet depleted groups (Figure 3). Platelet depletion or isotype antibody treatments were performed by a single injection with the appropriate preparation at 7 dpi and testing carried out at 9 dpi. In this test, the level of anxiety-like behavior is assessed in terms of the percentage of time spent in the open arms of the maze (Figure 3Ai,Aiii; Supplementary Table S2). There was no significant difference between vehicle-only/isotype antibody-treated and vehicle-only/platelet depleted groups in the percent time spent in the open arms of the maze (open arm duration (%), VO vs. VO + PD, 42.3 ± 7.2 vs. 41.8 ± 11.3, *p* = 0.974) showing that platelet depletion in the absence of EAE induction is not associated with anxiety. On the other hand, a significant difference between the above groups and the EAE-induced/isotype antibody-treated group was demonstrated, showing that EAE induction is associated with anxiety-like behavior (open arm duration (%), VO vs. EAE, 42.3 ± 7.2 vs. 20.7 ± 5.4, *p* = 0.036) and that this effect is already evident from the preclinical stage. However, there was a significant increase in the percent time spent in the open arms between the EAE-induced/isotype antibody-treated and EAE-induced/platelet depleted groups showing a beneficial effect of platelet depletion on anxiety-like behavior (open arm duration (%), EAE vs. EAE + PD, 20.7 ± 5.4 vs. 70.2 ± 8.1, *p* = 0.005). An additional important measure is that of total distance covered over the time of experimentation to identify potential early ambulatory difficulties, undetectable by visual observation, which may confound the test (Figure 3Aii; Supplementary Table S3). There was no significant difference in the total distance covered during the test period between groups, including vehicle groups (distance moved (cm), VO vs. VO + PD, 1090.7 ± 56.3 vs. 984.6 ± 68.6, *p* = 0.261), EAE groups (distance moved (cm), EAE vs. EAE + PD, 965.4 ± 66.4 vs. 828.5 ± 85.4, *p* = 0.222) or treatment groups (distance moved (cm), VO + PD vs. EAE + PD, 984.6 ± 68.6 vs. 828.5 ± 85.4, *p* = 0.212) showing absence of ambulatory difficulties at the time at which experimentation was conducted. At 9 dpi, all mice exhibited a clinical score of zero (Figure 3Ci) and there were no significant differences in percent weight change between groups (weight (%) from 0 dpi, EAE vs. EAE + PD, 97.6 ± 0.5 vs. 98.8 ± 0.2, *p* = 0.531) (Figure 3Cii).

Following testing in the EPM, half of the mice in each control and experimental group were immediately humanely killed and the dorsal hippocampal region dissected for total RNA extraction, generation of cDNA and qPCR analysis of the pro-inflammatory cytokines TNF-α and IFN-γ and the platelet specific marker CD41 (Figure 3Bi–Biii). In the case of TNF-α and IFN-γ, a significant difference was observed between the above groups and the EAE-induced/isotype antibody-treated group, showing that even by 9 dpi, a severe inflammatory environment was present in the hippocampal region. This pro-inflammatory environment was associated with the presence of platelets as shown by the significant difference in CD41 expression levels between the same groups. Platelet depletion resulted in the significant reduction in expression of the pro-inflammatory and platelet markers. As an additional control, the remaining mice in each group were maintained on the treatment assigned to their group and were humanely killed at 14 dpi. Only the EAE-induced/isotype antibody group developed EAE (clinical score of 2.0 ± 0.1 at 14 dpi, (Figure 3Ci), whilst vehicle-only/isotype antibody, vehicle-only/platelet depleted and EAE-induced/platelet depleted groups remained clinical score free, nor did they exhibit weight loss (weight (%) from 0 dpi, VO vs. EAE, 100 ± 0.1 vs. 83.9 ± 0.3, *p* < 0.001) (Figure 3Cii).

### 3.3. Anxiety-Like Behavior and the Pro-Inflammatory Environment in the Hippocampus are Characterized by Platelet–Neuron Association

**Figure 4:**
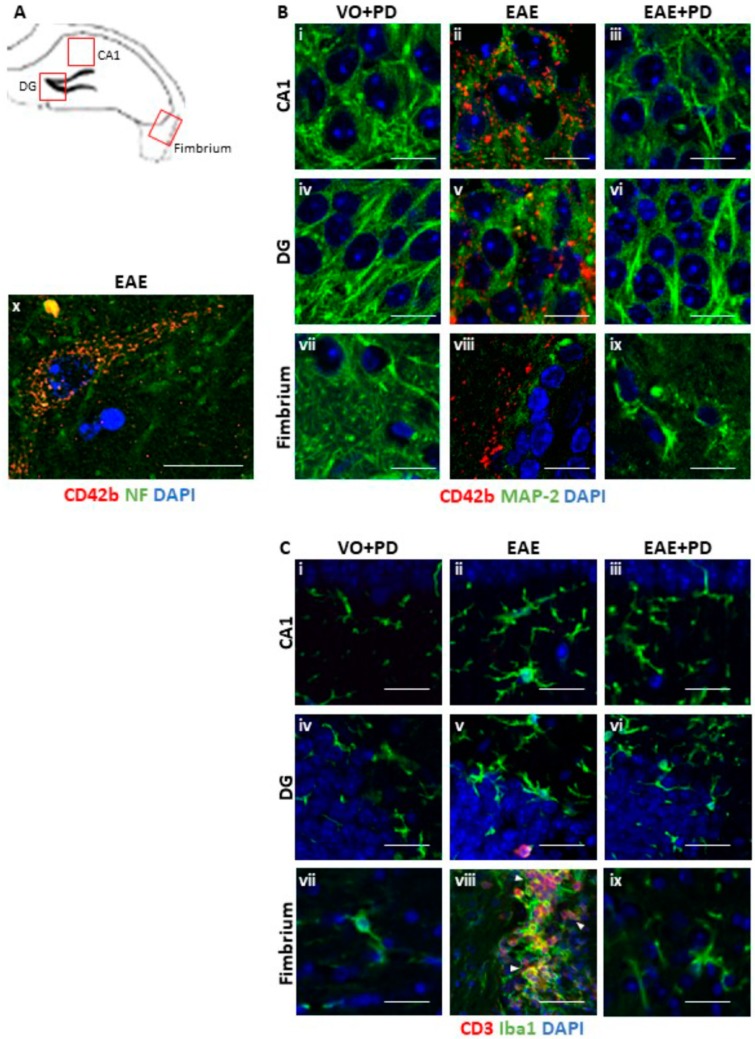
Immunochemical analysis of platelet accumulation and immune cell infiltration in the hippocampus. Following EAE induction, mice received a single administration of platelet depletion antibody (EAE + PD) or isotype control (EAE) at 7 dpi and were exposed to the EPM at 9 dpi, with *n* = 8 mice/group. An additional control consisted of PD-treated vehicle-only mice (VO + PD). Half of each cohort was killed at 9 dpi and the other at 14 dpi and sections (14 µm thick) challenged with anti-CD42b (red) + anti MAP-2 (green) (**Bi** to **Bix**), or anti-CD42b (red) + anti NF (green) (**Bx**), or anti-CD3 (red) + anti-Iba1 (green) (**C**). Nuclei were stained with DAPI. (**A**) shows the regions of interest. Platelets were observed in the fimbrium and in close association with neuronal cell bodies in the CA1 region and dentate gyrus (DG) only in EAE mice (**Bii**, **Bv**, and **Bviii**), but not in VO + PD (**Bi**, **Biv**, and **Bvii**) and EAE + PD mice (**Biii**, **Bvi**, and **Bix**) at 14 dpi (**Bi** to **Bix**) and 9 dpi (**Bx**). (**Bx**) shows a single platelet-associated neuron in the CA1 region in an EAE mouse at higher magnification. T lymphocyte infiltration was not observed in the CA1 region (**Ci**–**Ciii**) and dentate gyrus (**Civ**–**Cvi**) over the whole time course. It was evident in the fimbrium (**Cviii**), but not the CA1 region (**Cvii**) and dentate gyrus (**Cix**) by 14 dpi. Scale bars = 10 µm. EAE = experimental autoimmune encephalomyelitis, EPM = elevated plus maze, VO = vehicle only, PD = platelet depletion, MAP-2 = microtubule-associated protein 2, NF = neurofilament protein, DAPI = 4′,6 diamidino-2-phenylindole; CA1 = cornu ammonis region 1.

**Figure 5:**
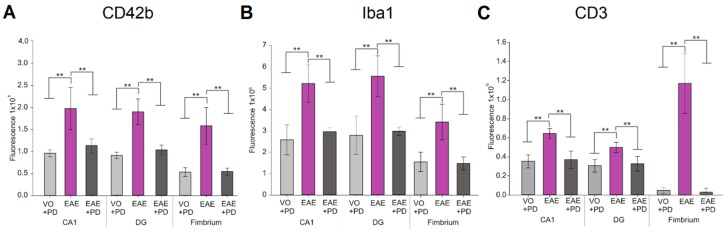
Quantification of platelet entry into the hippocampus and hallmarks of neuroinflammation. Immunofluorescence signals from each region shown in Figure 4 and from vehicle-only/platelet depleted (VO + PD), EAE-induced/isotype control-treated (EAE), EAE-induced/platelet depleted (EAE + PD) groups were captured and estimated by ImageJ using an arbitrary scale, with *n* = 4 mice/group × 6 sections/mouse. (**A**) CD42b (platelets), (**B**) Iba1 (microglial reactivity), and (**C**) CD3 (T lymphocytes) showed significantly higher expression in the EAE group relative to VO + PD and EAE + PD groups (** = *p* < 0.01). There was no significant difference between VO + PD and EAE + PD groups for all markers.

To further investigate the relationship between anxiety-like behavior, parenchymal platelet accumulation and lymphocytic infiltration, immunochemistry was performed with tissues from mice used in the EPM test (Figure 4). At 9 and 14 dpi, combined anti-CD42b and anti-MAP2 revealed extensive platelet accumulation in the EAE-induced/isotype antibody-treated group only, where they were particularly prominent in the CA1 region (Figure 4Bii), dentate gyrus (Figure 4Bv) and fimbrium (Figure 4Bviii). In the fimbrium, diffuse platelet distribution was observed; on the other hand, in the CA1 region and dentate gyrus platelets appeared to associate principally with neuronal cell bodies (Figure 4Bx). Quantification of immunofluorescence signals confirmed the significant difference in platelet accumulation between EAE-induced/isotype antibody-treated and EAE-induced/platelet depleted groups and absence of significance between EAE-induced/platelet depleted and vehicle-only/platelet depleted groups (Figure 5). Combined anti-Iba1 and anti-CD3 were used to identify parameters of inflammation relative to platelet accumulation. We have previously demonstrated significantly elevated Iba1 reactivity in the hippocampal formation in EAE, associated with minimal CD3 cell infiltration [41]. Here, Iba1 reactivity was also identified by larger cell bodies and more complex branching of processes throughout the whole of the dorsal hippocampus, in the EAE-induced/isotype antibody-treated group only (Figure 4Cii, Cv, Cviii), from 9 dpi. Again, as previously demonstrated, CD3 was identified only at 14 dpi and the presence of CD3 positive cells was restricted to the fimbrium and adjacent choroid plexus (Figure 4Cviii). Quantification of immunofluorescence signals confirmed the significant difference in Iba1 and CD3 levels between EAE-induced/isotype antibody-treated and EAE-induced/platelet depleted groups and absence of significance between EAE-induced/platelet depleted and vehicle-only/platelet depleted groups (Figure 5). Taken together, these data showed that the strong platelet presence in the hippocampal formation in the EAE/isotype antibody-treated group from the pre-clinical stage was not associated with inflammatory cell infiltration.

## 4. Discussion

A previous study from our laboratory showed a cause and effect relationship between platelet accumulation and subsequent clinical disease development [25]. Here, we confirm this relationship with (1) evidence of a temporal relationship between platelet accumulation in the circulation and that of MOG_35–55_-CD4^+^ cells; (2) the demonstration of a dependence for MOG_35–55_-CD4^+^ cell accumulation in the blood, lymphoid organs as well as the CNS, on prior platelet accumulation; and (3) absence of immune cell infiltration and clinical EAE with platelet depletion. MOG_35–55_-induced EAE is a T cell-driven disease in which CD4^+^ T cells dominate in lesions [49]. Taken together these data confirm the driving role of platelets in neuroinflammation, by promoting the generation of auto-aggressive T cells.

The establishment of the temporal relationship between platelet accumulation and MOG_35–55_-CD4^+^ cells also allowed for the identification of a time window for determination of a potential primary role for platelets in hippocampal functional deficits, using the EPM test. In this test there is a need to achieve treatment efficacy whilst minimizing potential confounding effects such as the stress arising from repeated iv injections of platelet depleting antibody, or the onset of ambulatory difficulties. From the ICS data, given that the earliest evidence of MOG_35–55_-CD4^+^ cells was between 10 and 12 dpi, we determined that the latest time point for performing the test in the absence of antigen-specific T cells was 9 dpi. Since platelet depletion at the dosage used is effective for 48 h, the latest timing for platelet depletion was therefore 7 dpi. Data showed that a single administration of platelet depletion antibody at 7 dpi was sufficient to demonstrate a significantly beneficial effect on anxiety-like behavior in terms of percentage of time spent in the open arms of the maze, relative to vehicle-only/platelet depleted animals. This effect was confirmed by the absence of platelets in the hippocampal formation by immunochemistry, supported by qPCR evaluation of the platelet-specific markers CD41, in the EAE-induced/platelet depleted group compared with control groups, as well as the significant reduction of levels of the pro-inflammatory cytokines TNF-α and IFN-γ. We do not believe that the effect of platelet depletion on anxiety-like behavior is a consequence of other changes, for example in levels of stress hormones. Firstly, in a previous study aimed at optimizing parameters for the EPM test in the EAE model [44], we found no treatment or induction effects on plasma corticosterone concentrations, and there was no significant correlation between the EPM test and plasma corticosterone concentrations. Secondly, we also demonstrated that platelet–neuron-association was co-incident with expression of platelet-specific pro-inflammatory molecules such as PF4 [25], which supports the notion of a direct deleterious effect of platelets on neurons.

The association between pro-inflammatory environment and neuropsychiatric symptoms in EAE is consistent with the literature, where a crucial role for pro-inflammatory cytokines was determined in behavioral deficits, prior to the appearance of motor symptoms. Using the EPM and the open field test (also a test of anxiety/depression), Haji et al. [50] demonstrated anxiety-like behavior by 7dpi, associated with upregulation of TNF-α in the striatum, a recognized major region for mood control. Additionally, disturbances in cognitive function from early disease have been identified by the novel object recognition test, associated with upregulation of Il-1β and IL-6 [51]. A significant observation in the present study was that neuropsychiatric symptoms in the EAE-induced/isotype antibody-treated group occurred in the presence of apparently intimate platelet–neuron association in the CA1 region and dentate gyrus, but the absence of CD3^+^ cells as shown by immunochemistry, or of CNS accumulation of MOG_35–55_-CD4^+^ cells by ICS. Immune cell infiltration was observed at the later time point of 14 dpi and was restricted to the fimbrium region, as previously reported by us [41]. From the sum of these observations we conclude that in EAE functional disturbance in the hippocampal formation begins in the pre-clinical disease stage, is related to platelet entry and associated generation of a pro-inflammatory environment, but not with inflammatory cell infiltration.

In a previous characterization of hippocampal inflammation in EAE, we demonstrated that at the peak of disease grey matter sub-regions of this complex structure were associated with minimal inflammatory cell infiltration, but significant microglial activation and expression of the CNS stress marker αB-crystallin [41]. In this communication, we also show early functional disturbance in this structure following EAE induction. These pathological features overlap with those described in the hippocampal formation in MS, by the suggestion of neuronal decline in the presence of paucity of inflammatory lesions. However, we also clearly demonstrate that hippocampal functional disturbances occur in the presence of platelet–neuron associations in EAE which are already evident prior to any measurable MOG_35–55_-CD4^+^ cells in the circulation or the CNS. Coupled with our previously reported similar observations in the spinal cord [25], the data provide proof of concept for an alternative mechanism which may underlie grey matter damage. They suggest that platelets participate in EAE pathophysiology by at least two mechanisms. First, platelet activation drives antigen-specific T cell accumulation as evidenced by the temporal relationship between platelet accumulation and that of CD4^+^ cells, together with absence of CD4^+^ cells and subsequent disease development following platelet depletion. Secondly, our collective evidence strongly suggests that platelets play a role in neurodegeneration which is independent of antigen-specific T cell accumulation. Thus, (1) platelet entry into the CNS parenchyma was identified prior to any detectable CD4^+^ cells in blood, lymphoid organs and the CNS and (2) platelet–neuron associations with concomitant generation of a pro-inflammatory environment and functional disturbance were demonstrated in regions where CD3 cells or lesions are never observed (retina) [25], or remain minimal (hippocampus and spinal cord grey matter) [25,41] over the disease trajectory. This scenario would also be consistent with the early occurrence of depression in MS, which often precedes presentation [36,37,38,39,40].

## 5. Conclusions

Platelets have long been implicated in MS pathogenesis [14,52]. Studies have described their enhanced adhesiveness in this condition and other abnormalities, including structural changes, upregulation of enzymes, and markers of platelet activation [19,20,21], as well as the presence of platelets in MS lesions [22]. More recently, it has become apparent that these abnormalities pertain not only to relapsing–remitting MS, but also to progressive disease forms together with clinically isolated syndrome [18], thereby highlighting platelet association with multiple disease forms and stages. This preliminary study was based on relatively small sample sizes (eight animals per group) together with a single behavioral test for hippocampal function. Despite these limitations, the data strongly suggest a mechanism for neurodegeneration in the absence of inflammatory infiltration, which is a hallmark of progressive MS phenotypes. Thus, future studies should focus on the relationship between platelets and disease progression and the potential of these elements as novel therapeutic targets for MS.

## Figures and Tables

**Table 1:** Target genes and their primer pairs.

Target Gene	Forward Primer Sequence (5′–3′)	Reverse Primer Sequence (5′–3′)
IFN-γ	TCATGGCTGTTTCTGGCTGT	CCCAGATACAACCCCGCAAT
TNF-α	AAGCCTGTAGCCCACGTCGTA	GGCACCACTAGTTGGTTGTCTTTG
CD41	TTTCTGCAGCCTAAGGGCC	GGCAGCCACAGCAATATCATT
HSP90	GCTTTCCCGTCAAGATGCCT	CACCACTTCCTTGACCCTCC
GAPDH	GCTCATGACCACAGTCCATGC	GTTGGGATAGGGCCTCTCTTG
β-actin	AGTGTGACGTTGACATCCGT	GCAGCTCAGTAACAGTCCGC

## Supplementary Materials

The following are available online at http://www.mdpi.com/2077-0383/8/2/162/s1, Table S1: The main effects and interaction effects of the two parameters under investigation, on plasma platelet counts per µL. Table S2: The main effects and interaction effects of the two parameters under investigation, on the percentage of total time spent in open arms of the EPM. Table S3: The main effects and interaction effects of the two parameters under investigation, on locomotor activity.
