# Supplementary material for: Platelet Depletion is Effective in Ameliorating Anxiety-Like Behavior and Reducing the Pro-Inflammatory Environment in the Hippocampus in Murine Experimental Autoimmune Encephalomyelitis
Source: J Clin Med. 2019 Feb 1;8(2):162. doi: 10.3390/jcm8020162 (PMC6406682; doi:10.3390/jcm8020162)
Supplement: Supplementary file 1 [file jcm-08-00162-s001.pdf]

Supplementary Tables

Supplementary Table S1. The main effects and interaction effects of the 2 parameters under investigation, on plasma platelet counts per μL.

2 × 2 Univariate ANOVA		Platelet Counts (Number/μL)
Main effects	Induction	F(1,16) = 1.338 , *p* = 0.264
	Treatment	F(1,16) = 6.317 , *p* =0.023
Interaction effect	Induction × Treatment	F(1,16) = 0.626 , *p* =0.441

The main effects (induction and treatment) and interaction effects between these parameters are shown in terms of platelet counts (×10^5/uL) in a 2 × 2 Univariate ANOVA. Statistical difference ($p \leq 0.05$) is found for the treatment effect.

Supplementary Table S2. The main effects and interaction effects of the 2 parameters under investigation, on the percentage of total time spent in open arms of the EPM.

2 × 2 Univariate ANOVA		Percent Open Arm Duration
Main effects	Induction	F(1,24) = 0.182 , *p* = 0.673
	Treatment	F(1,24) = 9.334 , *p* =0.005
Interaction effect	Induction × Treatment	F(1,24) = 9.667 , *p* =0.005

The main effects (induction and treatment) and interactions between these parameters are shown in terms of percent open arm duration in a 2 × 2 Univariate ANOVA. Statistical difference ($p \leq 0.05$) is found for the treatment effect.

Supplementary Table S3. The main effects and interaction effects of the 2 parameters under investigation, on locomotor activity.

2 × 2 Univariate ANOVA		Distance Moved (cm)
Main effects	Induction	F(1,24) = 3.886 , *p* = 0.060
	Treatment	F(1,24) = 2.897 , *p* = 0.102
Interaction effect	Induction × Treatment	F(1,24) = 0.046 , *p* = 0.831

The main effects (induction and treatment) and interactions between these parameters are shown in terms of distance moved (cm) in a 2 × 2 Univariate ANOVA. No statistical difference ($p \geq 0.05$) is found for the treatment effect.
